# mTORC2 Regulates Lipogenic Gene Expression through PPAR*γ* to Control Lipid Synthesis in Bovine Mammary Epithelial Cells

**DOI:** 10.1155/2019/5196028

**Published:** 2019-05-16

**Authors:** Zhixin Guo, Keyu Zhao, Xue Feng, Dandan Yan, Ruiyuan Yao, Yuhao Chen, Lili Bao, Zhigang Wang

**Affiliations:** ^1^State Key Laboratory of Reproductive Regulation & Breeding of Grassland Livestock, School of Life Sciences, Inner Mongolia University, Hohhot 010021, China; ^2^School of Life Sciences, Jining Normal University, Jining 012000, China; ^3^School of Basic Medical Science, Inner Mongolia Medical University, Hohhot 010110, China

## Abstract

The mechanistic target of rapamycin complex 2 (mTORC2) primarily functions as an effector of insulin/PI3K signaling to regulate cell proliferation and is associated with cell metabolism. However, the function of mTORC2 in lipid metabolism is not well understood. In the present study, mTORC2 was inactivated by the ATP-competitive mTOR inhibitor AZD8055 or shRNA targeting* RICTOR* in primary bovine mammary epithelial cells (pBMECs). MTT assay was performed to examine the effect of AZD8055 on cell proliferation. ELISA assay and GC-MS analysis were used to determine the content of lipid. The mRNA and protein expression levels were investigated by RT/real-time PCR and western blot analysis, respectively. We found that cell proliferation, mTORC2 activation, and lipid secretion were inhibited by AZD8055.* RICTOR* was knocked down and mTORC2 activation was specifically attenuated by the shRNA. Compared to control cells, the expression of the transcription factor gene* PPARG* and the lipogenic genes* LPIN1*,* DGAT1*,* ACACA*, and* FASN* was downregulated in* RICTOR* silencing cells. As a result, the content of intracellular triacylglycerol (TAG), palmitic acid (PA), docosahexaenoic acid (DHA), and other 16 types of fatty acid was decreased in the treated cells; the accumulation of TAG, PA, and DHA in cell culture medium was also reduced. Overall, mTORC2 plays a critical role in regulating lipogenic gene expression, lipid synthesis, and secretion in pBMECs, and this process probably is through PPAR*γ*. This finding provides a model by which lipogenesis is regulated in pBMECs.

## 1. Introduction

The solid matter of milk from dairy cows is composed primarily of proteins, fat, and lactose. Milk fat is the most variable component of milk—95% to 98% of which is triacylglycerol (TAG), with the remainder comprising other lipids [1, 2]. A complex network of proteins controls milk synthesis with mTOR (mechanistic target of rapamycin) functioning as a central regulator, and milk fat synthesis appears to be governed, at least in bovines, by an interactive network between PPAR*γ* and other transcription factors [3]. A transcriptome-wide analysis has revealed that the PPAR*γ*-coding gene* PPARG* is critical in milk fatty acid metabolism in goat mammary epithelial cells [4].

Numbers of the peroxisome proliferator-activated receptors (PPARs) family, such as PPAR*γ*, PPAR*α*, and PPAR*β*/*δ*, are a group of lipogenesis-related transcription factors and are critical in lipid metabolism during lactation—particularly, the PPAR*γ* network, which controls milk fat synthesis in lactating ruminants [5]. In the last 20 years, PPAR*γ* has been studied extensively in monogastrics and ruminants and is pivotal in controlling lipid metabolism [3, 5]. PPAR*γ* is activated by several long-chain fatty acids or the PPAR*γ* agonist, along with upregulating such target genes as* DGAT1* and* LPIN1 *[5, 6], which encode important enzymes in fatty acid esterification and TAG synthesis [7, 8], and* FASN* and* ACACA* [5, 6], which encode key enzymes to catalyze the synthesis of fatty acids [9]. The genes for the enzymes are overexpressed in Holstein dairy cows during lactation and promote milk fat synthesis and secretion [10].

mTOR combines with various components to form two mTOR complexes, mTORC1 and mTORC2, and integrates nutritional signals, growth factors, and energy status to regulate protein synthesis, cell growth, and metabolism [11]. mTORC1 (RAPTOR) is a central regulator of cell metabolism and is sufficient for the accumulation of triglycerides and* de novo *fatty acid biosynthesis [12–14], whereas the function of mTORC2 (RICTOR) in lipid metabolism is not well understood.

The most important achievement of studies on mTORC2 is likely the role in phosphorylation and activation of AKT/PKB, a key effector of insulin and growth factor signaling [11, 15, 16]. Recent findings have demonstrated a novel function of mTORC2 in regulating another group of lipogenesis-related transcription factors, sterol regulatory element-binding proteins 1 (SREBP1) [17, 18], but the function of mTORC2 on PPAR*γ* activation is not clear. Nevertheless, the regulatory function of mTORC2 on lipogenic gene expression via PPAR*γ* and the accumulation of triacylglycerol and fatty acids both extracellular and intracellular are unknown.

The purpose of this study was to determine the functions and mechanisms of mTORC2 in lipid biosynthesis and secretion by measuring the expression of* PPARG* and the lipogenic genes* LPIN1*,* DGAT1*,* ACACA*, and* FASN* in pBMECs. We propose a regulatory model of milk fat synthesis and secretion in bovine mammary epithelial cells, in which mTORC2 regulates lipogenic gene expression and milk lipid synthesis through PPAR*γ*. The results of this study provide insights into the precise mechanism by which lipid synthesis and secretion are regulated in mammary epithelial cells.

## 2. Materials and Methods

### 2.1. Primary Bovine Mammary Epithelial Cells Cultures

All animals and procedures used in this study were conducted according to the guidelines for the care and use of experimental animals established by the Inner Mongolia University Animal Care and Use Committee. Mammary tissues were obtained from 3 Chinese Holstein cows after being slaughtered in a commercial cattle slaughter farm. The primary bovine mammary epithelial cells (pBMECs) were isolated and cultured by the adherent culture of small-sized cow mammary tissues. After mammary tissues were surgically removed from the slaughtered cow, they were placed in sterile, ice-cold phosphate-buffered saline (PBS) that was supplemented with 300 U/mL penicillin G and 100 mg/mL streptomycin (V900929, Sigma-Aldrich, Inc. St. Louis, MO, USA) and transported immediately to the laboratory. The mammary tissues were trimmed of visible fat and connective tissue and washed with PBS several times until the solution became pellucid and devoid of milk. Then, the mammary tissues were cut into small pieces (approx. 1×1×1 mm^3^) and established as a primary culture, from which bovine mammary epithelial cells (BMECs) were isolated and cultured, and cell morphology was examined by light microscopy. Purified primary BMECs were maintained and incubated in DMEM/F12 media (SH30023.01, Hyclone Laboratories, Inc. Logan, UT, USA) that contained 10% fetal bovine serum (04-001-1ACS, Biological Industries, Israel) and supplemented with 5 *μ*g/ml insulin (I6634, Sigma-Aldrich, Inc. St. Louis, MO, USA), 10 ng/ml epidermal growth factor (EGF, E4127, Sigma-Aldrich, Inc. St. Louis, MO, USA), 1 *μ*g/ml hydrocortisone (G8450, Solarbio Life Sciences, Beijing, China), and 1 *μ*g/ml progesterone (V900699, Sigma-Aldrich, Inc. St. Louis, MO, USA) in a 25 cm^2^ tissue culture flask at 37°C in humidified air with 5% CO_2_ as described [19, 20]. The expression of* KRT8* (*keratin 8*),* KRT18* (*keratin 18*), and* CSN2* (*casein beta*) was examined by RT-PCR with the primers (Table S1); the proteins of KRT7 (keratin 7), KRT18 (keratin 18), and CSN2 were examined by immunofluorescence. P_2_ to P_4_ primary bovine mammary epithelial cells (pBMECs) that were in the logarithmic growth phase were used to all experimental assays. The bovine mammary fibroblasts were also isolated and cultured by the adherent culture of small-sized cow mammary tissue, and the purified bovine mammary fibroblasts were used as negative control cells for detecting* CSN2 and VIM (vimentin*) by RT-PCR with the primers (Table S1) and examination of CSN2 and VIM (vimentin) by immunofluorescence.

### 2.2. Reagents and Antibodies

AZD8055, an ATP-competitive mTOR inhibitor, was purchased from Selleck Chemicals (S1555, Selleck Chemicals, 9330 Kirby Drive, STE 200m Houston, TX 77054, USA) and dissolved in DMSO (D2650, Sigma-Aldrich, Inc. St. Louis, MO, USA). The stock concentration is 10 mM. The proportion of DMSO in the cell culture medium is less than 0.5% (v/v) in any experiment.

The following primary antibodies were used in this study: anti-p-S6 (Ser240/244,** #**5346s), anti-AKT (**#**9272), anti-ACC (**#**3662), anti-p-AKT (Ser473,** #**9271s), anti-p-4EBP1 (Thr37/46,** #**2855s) (Cell Signaling Technology, Inc., Beverley, MA, USA); anti-S6 (ab184551), anti-4EBP1 (ab2606), anti-PPAR*γ* (ab45036), anti-LPIN1 (ab70138), anti-p-mTOR (Ser2448, ab32028), anti-mTOR (ab10926), anti-DGAT1 (ab100982), anti-RICTOR (ab105479), anti-FAS (ab22759) (Abcam, plc 330 Cambridge Science Park, Cambridge, UK); anti-*β*-actin (A5441, Sigma-Aldrich, Inc. St. Louis, MO, USA); ECL Anti-Rabbit IgG-HRP (NA934-100 *μ*l) and ECL Anti-Mouse IgG-HRP (NA931-100 *μ*l) (GE Healthcare, Little Chalfont, Buckinghamshire, UK).

### 2.3. MTT Assay

Procedures were performed as we described before [21]. Briefly, exponentially growing pBMECs were seeded into 96-well plates at 6×10^3^ cells per well 24 h before drug treatment. Then cells were treated with AZD8055 at various concentrations (1.5625 nM, 3.125 nM, 6.25 nM, 12.5 nM, 25 nM, 50 nM, 100 nM, and 200 nM) for 12 h and 24 h. MTT absorbance was measured with a spectrophotometer set (Thermo, Multiskan SX 353, USA) to evaluate the inhibitory efficiency of inhibitor on cell proliferation.

### 2.4. DNA Construct and In Vitro Transfection

A short hairpin RNA (shRNA) (5′-aaGTACAGAATTGCTACTAGGTTCAAGAGACCTAGTAGCAATTCTGTACtt-3′) that harbored the siRNA that targets* RICTOR* was designed based on the sequence of bovine* RICTOR* gene (NM_001144096.3). A double-stranded DNA fragment was generated by chemical synthesis, encoding the* RICTOR*–shRNA with a* Bam*HI restriction site at the 5′ end and a* Hin*dIII restriction site at the 3′ end. The DNA fragment was inserted into the multiple cloning site of pRNAT–U6.1/Neo plasmid, which is a GenScript siRNA expression vector (Cat. No. SD1211, GenScript Biotech Corp, Nanjing, China), to yield pRNAT-U6.1/Neo-RICTOR-shRNA. The plasmid pRNAT-U6.1/Neo-shRICTOR was transfected into primary BMECs using Lipofectamine TM2000 (11668019, Invitrogen, Carlsbad, New Mexico, USA) per the manufacturer's instructions. Transfected cells were selected with G418 (SV30068.02, Hyclone Laboratories, Inc. Logan, Utah, USA) for 48 h and imaged under a digital fluorescence microscope (Carl Zeiss Microscopy, LLC One Zeiss Drive, Thornwood, NY 10594, USA), and then cell culture medium and cells were separated and collected, respectively.

### 2.5. ELISA

ELISA was used to detect the concentration of triacylglycerol (TAG), palmitic acid (PA), and docosahexaenoic acid (DHA) as previously described [21]. Primary BMECs were seeded into 6-well plates at 8×10^5^ cells per well, incubated until 80% confluence. For the inhibitor experiments, cells were treated with 100 nM AZD8055 for 12 h or 24 h; for the* RICTOR* knocking down experiments, cells were transfected with pRNAT-U6.1/Neo-shRICTOR and the transfected cells were selected with G418 for 48 h. Cell culture medium was collected for measurement of extracellular TAG, PA, and DHA. Control and treated primary BMECs were harvested with trypsin and were centrifuged to remove supernatants, and then cell lysates were prepared. Equal volume of protein lysates was measured for TAG, PA, and DHA by ELISA. All measurements were made in triplicate, and the mean values of at least 3 repeat experiments were used for the statistical analysis.

### 2.6. Western Blot Analysis

Western blot was used to detect the expression of indicated proteins and phosphorylated proteins as previously described [22]. Briefly, control and treated primary BMECs were harvested with trypsin and lysed in cell lysis buffer. Equal amounts (40 *μ*g) of protein were electrophoresed, and transferred to polyvinylidene fluoride membranes, and incubated with the primary antibody. Peroxidase-conjugated secondary antibody and enhanced chemiluminescence (ECL) reagent were used to detect the signals with the Western Blotting System.

### 2.7. qPCR Analysis

The quantitative real-time polymerase chain reaction (qPCR) was performed according to Guo and Wang [20] with some modifications. RT-qPCR was used to determine the mRNA abundance of* PPARG*,* DGAT1*,* LPIN1*,* ACACA, FASN, *and* RICTOR* in the primary BMECs of the treatment groups and control. Cells were transfected with pRNAT-U6.1/Neo-shRICTOR and the transfected cells were selected with G418 for 48 h. Total RNA was isolated from the untreated and treated cells using RNAzol (9109, TaKaRa Co. Ltd., Dalian, China), following the manufacturer's instructions. RNA quantities over 600 ng/*μ*L and a purity of 1.90~2.0 by as 260/280 ratio 1.90~2.0 were used to synthesize cDNA. The RNA integrity was assessed by electrophoresis.

Total RNA was reverse-transcribed with an oligo (dT)_12–18_ primer using the* EasyScript*® One-Step gDNA Removal and cDNA Synthesis SuperMix Kit (AE311, TransGen Biotech Co. Ltd., Beijing, China), and gDNA was removed. cDNA sequences were amplified with the primers shown in Table 1.* GAPDH*,* RPS15A*,* PPIA*,* ACTB*, and* B2M* were tested as internal control genes, and* ACTB* was selected as the best internal control gene (Table S2), according to available NormFinder and the stability values [23]. The target genes that were selected for evaluation in RNA samples from control and treated cells were related to lipid synthesis (i.e.,* PPARG*,* DGAT1*,* LPIN1*,* ACACA*, and* FASN*) (Table 1). All primer pairs were designed with Primer Premier Software (PREMIER Biosoft) and confirmed using Primer-BLAST (NCBI) online. The presence of a single product and the absence of primer dimers were verified by agarose gel electrophoresis.

The reactions were run using the KAPA SYBP® FAST qPCR Kit Optimized for LightCycler® 480 (KM4110, KAPA BIOSYSTEMS, Inc., Boston, Massachusetts, USA) according to the manufacturer's instructions. Three technical replicates were run. 2^−ΔΔCT^ values were calculated to determine expression levels, and the qPCR results were analyzed by Student's t-test to compare the expression between untreated and treated groups. 3 independent experiments were performed.

### 2.8. Gas Chromatography and Mass Spectrum

Cells were transfected with pRNAT-U6.1/Neo-RICTOR-shRNA and the transfected cells were selected with G418 for 48 h. Untreated and treated cells were collected and dissolved in 1 mL lysis buffer. Fatty acid methyl esters (FAME) were extracted twice with n-hexane at room temperature and evaporated to dryness at 30°C for 30 min, then dissolved in n-hexane, and then separated in a gas chromatography-mass spectrum (Shimadzu, GCMS-QP2010 ultra, Shimadzu, Japan) using an Agilent HP-88 capillary-column (100 m × 0.25 mm × 0.20 *μ*m, Agilent Technologies, Santa Clara, CA, USA). The program was set to column temperature 60°C for 1 min, with ramping of 40°C/min up to 140°C, and a hold for 10 min, 4°C/min up to 240°C, and a hold for 15 min. The injector temperature was 220°C, and the sample was 1 *μ*L. The injection mode was split flow. External standards were obtained from Sigma-Aldrich (Cat. No.18919-1AMP).

### 2.9. Statistical Analyses

Data are presented as mean ± SD. Statistical significance for the dose effect was determined by a general linear model, the percentage of fatty acid was determined by chi-square, and other data were determined by one-way ANOVA, followed by Tukey's method. Statistical analyses were conducted using SPSS PASW Statistics for Windows, v18.0 (SPSS Inc.: Chicago, IL, USA). The western blot results were quantified on a Gel-Pro Analyzer 4.0 (Media Cybernetics, USA). The results were presented as the average of at least 3 biological replicates.* p*≤ 0.05 was considered to be statistically significant.

## 3. Results

### 3.1. AZD8055 Inhibits Proliferation of pBMECs

Primary bovine mammary epithelial cells (pBMECs) were isolated by the adherent culture of small-sized cow mammary tissues, and their morphology was of a typical epithelial cell. The biomarker genes* KRT18*,* KRT8, *and* CSN2 *were transcribed, and the biomarker proteins KRT7, KRT18, and CSN2 were expressed in pBMECs, whereas the biomarker gene* CSN2* and protein CSN2 were not expressed in the negative control cells, bovine mammary fibroblasts (Figure S1).

**Figure 1:**
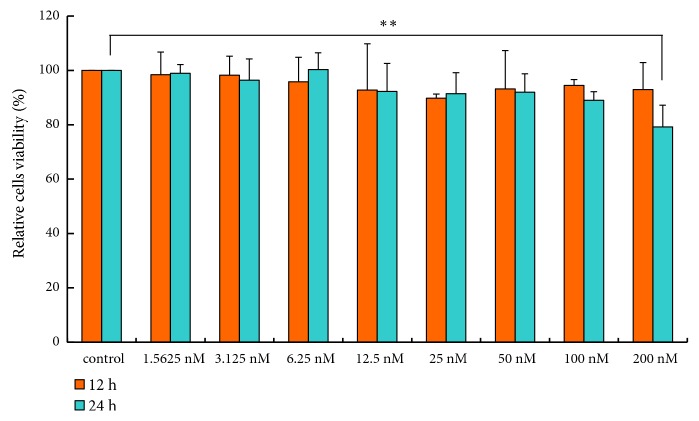
AZD8055 inhibits cell proliferation of primary bovine mammary epithelial cells (pBMECs). AZD8055 inhibits cell proliferation of pBMECs when its concentration is 200 nM for 24 h.* p*=0.001, *∗p*<0.01; n=3 biological replicates. Error bars indicate SD.

AZD8055, an ATP-competitive mTOR inhibitor, prevents the phosphorylation of mTOR and then suppresses mTORC1 and mTORC2 simultaneously, and inhibits the phosphorylation of mTORC1 substrates S6 and 4E-BP1 as well as phosphorylation of the mTORC2 substrate AKT [24]. To ensure that the lipid levels in the culture medium were not affected by the difference in cell number between the control and treatment groups, we first confirmed that the inhibitor concentration and incubation time had no significant effect on cell number. We determined the effects of AZD8055 on pBMEC proliferation by MTT assay and observed that it inhibited cell proliferation at a concentration of 200 nM for 24 h (Figure 1) (*p*<0.05). Thus, we treated cells for 24 h with 100 nM AZD8055 in the subsequent experiments.

### 3.2. AZD8055 Attenuates Accumulation of Lipids in the Culture Medium and the Activation of mTORC1 and mTORC2 in pBMECs

**Figure 2:**
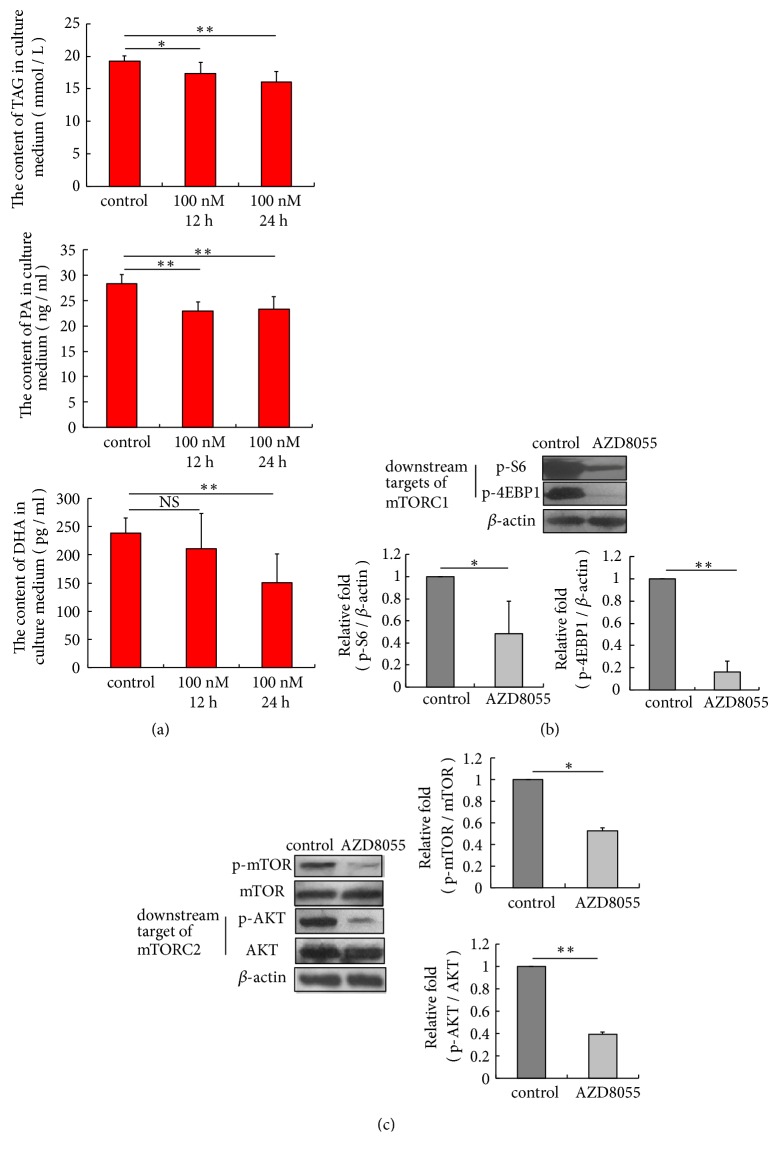
AZD8055 inhibits mTORC1 and mTORC2 activation simultaneously and decreases the accumulation of TAG, PA, and DHA in the culture medium. (a) Cells were treated with 100 nM AZD8055 for 12 h or 24 h and the content of TAG (*p*=0.031, p=0.001), PA (*p*=0.0001,* p*=0.0002), and DHA (*p*=0.335,* p*=0.006) was measured by ELISA. AZD8055 significantly decreased the accumulation of TAG, PA, and DHA in the culture medium. (b) AZD8055 attenuates the phosphorylation of downstream targets of mTORC1, including S6 (Ser240/244), and 4EBP1 (Thr37/46). (c) AZD8055 attenuates the phosphorylation of mTOR (Ser2448) and downstream target of mTORC2, AKT (Ser 473). *∗p*<0.05, *∗∗ p*<0.01, ns, no significantly; n=3 biological replicates. Error bars indicate SD.

To determine the effects of AZD8055 on lipid secretion, we treated pBMECs with 100 nM AZD8055 for 24 h, and the content of TAG, PA, and DHA in the culture medium was determined. The results showed that AZD8055 inhibited the secretion of TAG, PA, and DHA (Figure 2(a)) (*p*<0.01). Further, we determined the activation of mTORC1 and mTORC2, and the results showed that the phosphorylation of S6 (Ser240/244) and 4EBP1 (Thr37/46) was inhibited (Figure 2(b)), indicating that mTORC1 signaling was impeded by AZD8055. Also, the phosphorylation of mTOR (Ser2448) and AKT (Ser 473), a direct substrate of mTORC2, was attenuated by the inhibitor (Figure 2(c)), suggesting that mTORC2 signaling is also inhibited by AZD8055. We concluded that AZD8055 attenuated secretion, and mTORC2 is associated with secretion and synthesis of lipid in pBMECs.

### 3.3. AZD8055 Decreases the Protein Level of Intracellular PPAR*γ* and LPIN1, DGAT1, ACC, and FAS in pBMECs

**Figure 3:**
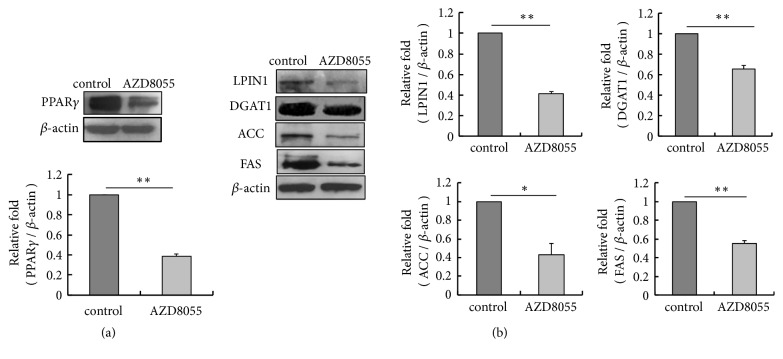
AZD8055 inhibits the protein level of intracellular PPAR*γ*, LPIN1, DGAT1, ACC, and FAS in pBMECs. (a) The protein level of intracellular transcription factor PPAR*γ* was inhibited by AZD8055. (b) The protein level of intracellular enzymes LPIN1, DGAT1, ACC, and FAS was inhibited by AZD8055. *∗p*<0.05, *∗∗ p*<0.01, n=3 biological replicates. Error bars indicate SD.

The experiments above demonstrated that AZD8055 attenuates accumulation of TAG, PA, and DHA in the culture medium and the activation of mTORC1 and mTORC2; thus, we speculated that the expression of lipogenesis related transcription factor PPAR*γ* and enzymes related to lipid synthesis is inhibited by AZD8055. We examined the level of intracellular PPAR*γ*, LPIN1, DGAT1, ACC, and FAS by western blot. The results showed that the protein level of intracellular PPAR*γ* and these catalyzing enzymes was decreased by the inhibitor (Figures 3(a) and 3(b)), indicating mTORC2 is associated with the expression of transcription* PPARG* and the lipogenic genes.

### 3.4. Inactive mTORC2 Downregulates the Expression of PPARG and Lipogenic Genes in pBMECs

**Figure 4:**
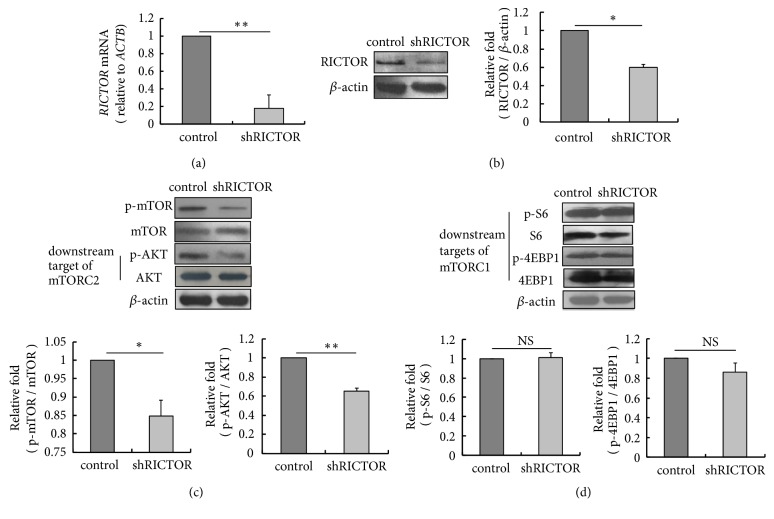
*RICTOR* silencing decreases mTORC2 activation but not mTORC1 in pBMECs. (a)* RICTOR* mRNA level is decreased in primary bovine mammary epithelial cells transfected with pRNAT-U6.1/Neo-RICTOR-shRNA (*p*=0.01). (b) RICTOR protein level is decreased in primary bovine mammary epithelial cells transfected with pRNAT-U6.1/Neo-RICTOR-shRNA. (c)* RICTOR* silencing attenuates the phosphorylation of mTOR (Ser2448) and downstream target of mTORC2, AKT (Ser 473). (d)* RICTOR* silencing does not attenuate the phosphorylation of downstream targets of mTORC1, including S6 (Ser240/244), and 4EBP1 (Thr37/46). *∗p*<0.05, *∗∗ p*<0.01, ns, no significantly; n=3 biological replicates. Error bars indicate SD.

Now that the data above demonstrated that AZD8055 inhibited the mTORC2 activation and the protein level of intracellular transcription factor PPAR*γ*, and enzymes-LPIN1, DGAT1, ACC, and FAS in pBMECs, we have reason to speculate that the expression of* LPIN1*,* DGAT1*,* ACACA*, and* FASN* is regulated by mTORC2 via PPAR*γ*. To evaluate whether mTORC2 can regulate expression of* PPARG *and* LPIN1*,* DGAT1*,* ACACA*, and* FASN*, the degree of mTORC2 activation was reduced by knocking down* RICTOR*, a critical component of mTORC2, using targeting shRNA in pBMECs. The transfected cells were incubated with G418 supplementing insulin and EGF for 48 h and imaged (Figure S2). We first examined the mRNA and protein expression levels and found* RICTOR* was knocked down at both levels (Figures 4(a) and 4(b)). The activity of mTORC1 and mTORC2 was detected by western blot. The results showed that phosphorylation of mTOR (Ser2448) and AKT (Ser473) was attenuated in* RICTOR* silencing cells (Figure 4(c)) while mTORC1 activation was not affected (Figure 4(d)). These data indicate mTORC2 activation was specifically inhibited by targeting shRICTOR but not of mTORC1 in pBMECs.

**Figure 5:**
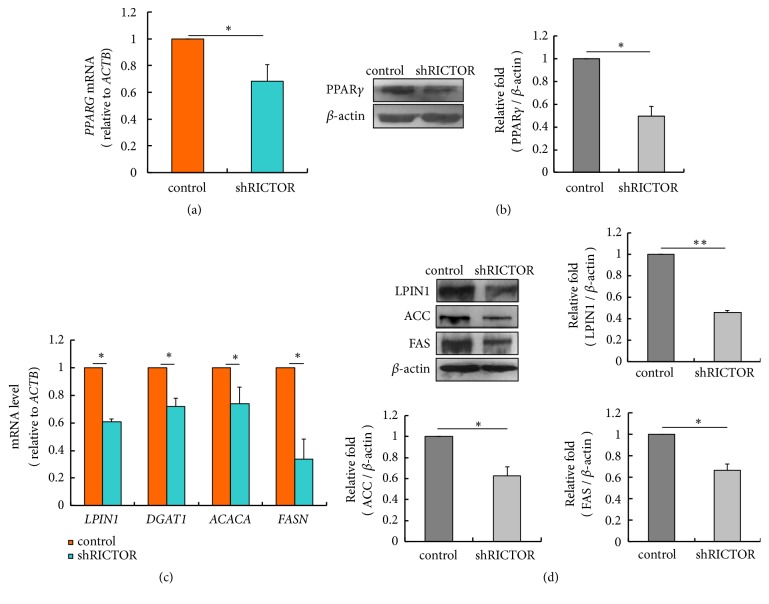
*RICTOR* silencing decreases the expression of* PPARG *and* LPIN1*,* DGAT1*,* ACACA *and* FASN* in pBMECs. (a)* RICTOR* silencing decreased* PPARG *mRNA abundance (*p*=0.023). (b)* RICTOR* silencing decreased PPAR*γ* protein level. (c)* RICTOR* silencing decreased the mRNA abundance of* LPIN1 *(*p*=0.025)*, DGAT1 *(*p*=0.017)*, ACACA *(*p*=0.022), and* FASN *(*p*=0.024). (d)* RICTOR* silencing decreased the protein level of intracellular PPAR*γ*, LPIN1, ACC, and FAS. *∗p*<0.05, *∗∗ p*<0.01, ns, no significantly; n=3 biological replicates. Error bars indicate SD.

Next, the mTORC2 signaling, which controls regulation of lipogenic gene expression via PPAR*γ*, was characterized. To this end, the mRNA levels of* PPARG*,* LPIN1*,* DGAT1*,* ACACA, *and* FASN* were measured by RT-qPCR, and the corresponding proteins were detected by western blot in control and* RICTOR *silencing cells. Inactive mTORC2 significantly decreased* PPARG *expression in mRNA level (Figure 5(a)) (*p*<0.05) and protein level (Figure 5(b)). Also,* LPIN1*,* DGAT1*,* ACACA, FASN *mRNA abundances (Figure 5(c)) (*p*<0.05) and intracellular protein levels (Figure 5(d)) were decreased. These data indicate that* RICTOR* silencing decreases the expression of these lipogenic genes, and this process probably is through PPAR*γ*.

### 3.5. Inactive mTORC2 Inhibits the Synthesis and Secretion of Triacylglycerol and Fatty Acids in pBMECs

**Figure 6:**
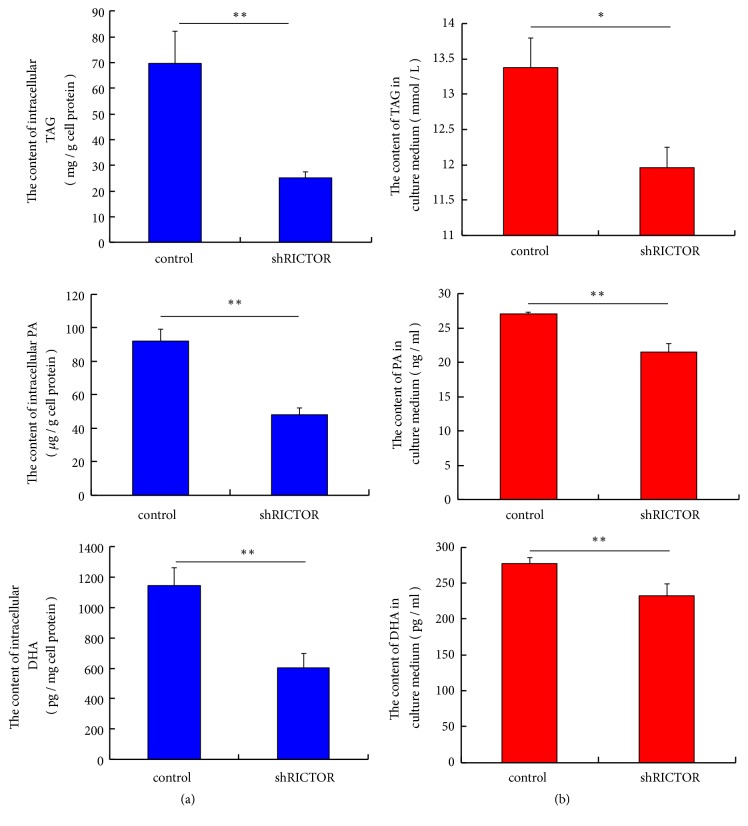
*RICTOR* silencing decreases the synthesis and secretion of lipid in pBMECs. (a)* RICTOR* silencing decreases the intracellular contents of TAG (*p*=0.008), PA (*p*=0.002), and DHA (*p*=0.007). (b)* RICTOR* silencing decreases the accumulation of TAG (*p*=0.018), PA (*p*=0.003), and DHA (*p*=0.01) in cell culture medium. *∗p*<0.05, *∗∗ p*<0.01, ns, no significantly; n=3 biological replicates. Error bars indicate SD.

To confirm TAG and fatty acid synthesis and secretion are regulated by mTORC2 in pBMECs, we measured intracellular content and extracellular content in cell medium of TAG, PA, and DHA by ELISA. Comparing to control cells, the intracellular content of TAG, PA, and DHA was decreased (Figure 6(a)) (*p*<0.01) in* RICTOR* silencing cells, and accumulation of these lipids in the culture medium was also significantly reduced (Figure 6(b)) (*p*<0.05). Further, a total of 24 types of intracellular fatty acid were assayed by GC-MS—18 decreased, including PA and DHA, and 6 rose, and total content declined in* RICTOR* silencing cells (Table 2). These data indicate that synthesis and secretion of TAG and several types of fatty acid are governed by mTORC2 in pBMECs.

## 4. Discussion

mTORC2 function in lipogenesis through SREBP1 in response to insulin has been established [17, 25, 26], but it is unknown whether mTORC2 is involved in* PPARG* expression. In this study, we examined the function of mTORC2 in* PPARG* expression. We found that knockdown of* RICTOR*/mTORC2 by shRNA downregulated* PPARG* expression and in turn decreased the expression of several lipogenic genes, including* LPIN1*,* DGAT1*,* ACACA*, and* FASN*. This finding implicates a novel function for mTORC2 which regulates the expression of lipogenic genes through PPAR*γ* to govern lipogenesis* in vitro*.

It is essential for reliable RT-qPCR to be able to normalize the data using internal control genes [27]. According to the minimum information for publication of quantitative real-time PCR experiments (MIQE) guidelines, at least 2 internal control genes were needed, unless there is a strong proof that the single reference gene used is indeed not affected by the treatment and can be a valid normalizer. The optimal number and choice of reference genes must be experimentally determined [28]. In the present study, five internal control genes were experimentally examined, and* ACTB* was selected as the best single internal control gene. The reference gene* ACTB* is a valid normalizer and was used as single reference gene in studies of vertebrate gene expression [29]. However, it is critical to assess the reliability of the normalization by testing multiple internal control genes [27], and the use of one internal reference gene has potential limitations in normalization. Consequently, the possibility that RT-qPCR data may be poorly normalized should be recognized in our experiments.

Epithelial cells are the central component of mammary alveoli, which produce milk during lactation. A lactating ruminant mammary cell model is useful for the study of milk synthesis. In recent years, significant results have been obtained from bovine and goat mammary epithelial cells, including the function of mTORC1 in cell proliferation, milk synthesis, and secretion [30–32] and that of lipogenesis-related transcription factors and lipogenic genes in lipid biosynthesis [33–35]. But little has been reported on the regulatory function of mTORC2 with regard to lipogenic gene expression and the lipid synthesis in bovine mammary epithelial cells. In our study, we found that mTORC2 is critical for the expression of* PPARG*,* LPIN1*,* DGAT1*,* ACACA*, and* FASN* and the biosynthesis of TAG and FAs. mTORC2 controls lipid synthesis and secretion in pBMECs.

In conclusion, in this study we have examined the function of mTORC2 in lipid biosynthesis and secretion in primary bovine mammary epithelial cells (pBMECs). mTORC2 plays a critical role in regulating lipogenic gene expression, lipogenesis, and secretion in pBMECs, and this process is through PPAR*γ*. This finding provides a model by which lipogenesis is regulated in pBMECs.

## Figures and Tables

**Table 1:** The target genes and primers for qPCR.

Gene Name	Primer sequence (5′→3′)
*RICTOR *(NM_001144096.3)	P1:CCCGACAGTATGTACGAGC
	P2: ATCCCAGAGTTTCCAGGTT
*PPARG* (NM_181024.2)	P1:GACGGGAAAGACGACAGACAAATC
	P2: CGTTCAAGTCAAGGTTCACAAAG
*DGAT1* (XM_025001413.1)	P1:AAGCCCTTCAAGGACATG
	P2:GCAGTAGAAGAAGATGAGCC
*LPIN1* (NM_001206156.2)	P1:AAAGTGAGCCAAAACGGGT
	P2:TTCTTTTCGATCACTTCCCTG
*ACACA *(NM_174224.2)	P1:ATCCCACGCATCTATGTAG
	P2:AGCACTGACTCTCTTGTAATC
*FASN *(NM_001012669.1)	P1:GGACGCTTTCCGTTACAT
	P2:CCAGTGATGATGTAGCTCTTG
*ACTB *(NM_173979.3)	P1:CACCACGGCCGAGCGGGAAATC
	P2:AGAGCCTCAGGGCAGCGGAACC

**Table 2:** Content of 24 types of intracellular fatty acid in control cells and *RICTOR*-silencing cells (%).

Fatty acid	Control^a^	shRICTOR^b^
Butyric acid (4:0)	0.81%	0.57% (*p*=1.00)
Lauric acid (12:0)	0.19%	0.12% (*p*=1.00)
Myristic acid (14:0)	1.14%	0.28% (*p*=0.22)
Pentadecanoic acid (15:0)	0.54%	0.08% (*p*=0.25)
cris-10-Pentadecenoic acid (15:1)	3.04%	0.68%*∗∗* (*p*=0.01)
Palmitic acid (16:0)	10.33%	5.62%*∗∗* (*p*=0.01)
Heptadecanoic acid (17:0)	1.04%	0.39% (*p*=0.45)
Stearic acid (18:0)	32.60%	19.82%*∗∗* (*p*=0.0001)
Linolelaidic acid (18:2n6t)	2.05%	1.15% (*p*=0.30)
Linoleic acid (18:2n6c)	5.39%	3.95% (*p*=0.36)
cis-11-Eicosenoic acid (20:1n9)	3.89%	2.03% (*p*=0.13)
cis-11,14,17-Eicosatrienoic acid (20:3n3)	3.38%	1.71% (*p*=0.15)
Eicosapentaenoic acid (20:5n3)	3.44%	2.37% (*p*=0.44)
Behenic acid (22:0)	0.95%	0.68% (*p*=0.73)
Erucic acid (22:1n9)	8.79%	1.93%*∗∗* (p=0.0001)
cis-13,16-Docosadienoic acid (22:2)	3.51%	2.61% (*p*=0.45)
Docosahexaenoic acid (22:6n3)	4.17%	2.22% (*p*=0.14)
Lignoceric acid (24:0)	0.85%	0.50% (*p*=0.69)
cris-10- Heptadecenoic acid (17:1)	3.28%	4.49% (*p*=0.41)
cis-11,14-Eicosadienoic acid (20:2)	2.41%	4.31% (*p*=0.14)
Arachidonic acid (20:4n6)	4.23%	10.39%*∗∗* (p=0.0001)
Henicosanoic acid (21:0)	0.66%	0.84% (*p*=1.00)
Tricosanoic acid (23:0)	0.57%	0.73% (*p*=1.00)
Nervonic acid (24:1n9)	2.76%	3.44% (*p*=0.71)
Total	100%	70.93%*∗∗* (p=0.0001)

a: content of product in control group/total content in control group × 100%.

b: content of product in *RICTOR*-silencing group/total content in control group × 100%.

shRICTOR: *RICTOR*-silencing (*∗∗p*<0.01).

## Data Availability

The data used to support the findings of this study are included within the article.

## Supplementary Materials

Supplementary Materials Supplementary File 1: Isolation and characterization of primary bovine mammary epithelial cells. Table S1: the biomarker genes and primers for PCR. Figure S1: morphology, biomarker genes, and biomarker proteins in primary bovine mammary epithelial cells. (a) Primary bovine mammary epithelial cells isolated by the adherent culture of small-sized cow mammary tissue for 7 days. (b) Purified bovine mammary epithelial cells. (c) Purified bovine mammary fibroblasts. (d) Specific genes transcribed in pBMECs. M: DL2000 Marker; PCR products pg 1: of* KRT18* (504 bp); 2:* KRT8* (540 bp); and 3:* CSN2* (303 bp). (e) KRT7, KRT18, and CSN2 expressed in pBMECs. (f)* VIM* transcribed, and* CSN2* not in bovine mammary fibroblasts. M: DL2000 Marker; PCR products pg 1: of* VIM* (536 bp); 2:* CSN2*. (g) VIM expressed, and CSN2 not in bovine mammary fibroblasts. Supplementary File 2. Table S2: the internal control genes and primers for qPCR. Figure S2:* RICTOR* knocked down using targeting shRNA. (a) Control cells. (b) pRNAT-U6.1/Neo-RICTOR-shRNA-transfected pBMECs expressing the green fluorescent protein (EGFP). Scale bar: 200 px. (c) RNA integrity assessed via electrophoresis. Total RNA was isolated from control cells and* RICTOR* silencing cells, respectively. RNA quantity of control cells was 883.5 ng/*μ*L, and the* RICTOR* silencing cells were 683.4 ng/*μ*L. The RNA stock solution was diluted 10 times and then taken 2 *μ*L for electrophoresis. Total RNAs pg M: DL2000 Marker; 1: of control cells; 2:* RICTOR* silencing cells.
