# Supplementary material for: mTORC2 Regulates Lipogenic Gene Expression through PPARγ to Control Lipid Synthesis in Bovine Mammary Epithelial Cells
Source: Biomed Res Int. 2019 May 16;2019:5196028. doi: 10.1155/2019/5196028 (PMC6541957; doi:10.1155/2019/5196028)
Supplement: Supplementary Materials — Supplementary File 1: Isolation and characterization of primary bovine mammary epithelial cells. Table S1: the biomarker genes and primers for PCR. Figure S1: morphology, biomarker genes, and biomarker proteins in primary bovine mammary epithelial cells. (a) Primary bovine mammary epithelial cells isolated by the adherent culture of small-sized cow mammary tissue for 7 days. (b) Purified bovine mammary epithelial cells. (c) Purified bovine mammary fibroblasts. (d) Specific genes transcribed in pBMECs. M: DL2000 Marker; PCR products pg 1: of KRT18 (504 bp); 2: KRT8 (540 bp); and 3: CSN2 (303 bp). (e) KRT7, KRT18, and CSN2 expressed in pBMECs. (f) VIM transcribed, and CSN2 not in bovine mammary fibroblasts. M: DL2000 Marker; PCR products pg 1: of VIM (536 bp); 2: CSN2. (g) VIM expressed, and CSN2 not in bovine mammary fibroblasts. Supplementary File 2. Table S2: the internal control genes and primers for qPCR. Figure S2: RICTOR knocked down using targeting shRNA. (a) Control cells. (b) pRNAT-U6.1/Neo-RICTOR-shRNA-transfected pBMECs expressing the green fluorescent protein (EGFP). Scale bar: 200 px. (c) RNA integrity assessed via electrophoresis. Total RNA was isolated from control cells and RICTOR silencing cells, respectively. RNA quantity of control cells was 883.5 ng/μL, and the RICTOR silencing cells were 683.4 ng/μL. The RNA stock solution was diluted 10 times and then taken 2 μL for electrophoresis. Total RNAs pg M: DL2000 Marker; 1: of control cells; 2: RICTOR silencing cells. [file 5196028.f1.pdf]

Supplementary File 1 Isolation and characterization of primary bovine mammary epithelial cells

1. Materials and methods

1.1 Isolation of primary bovine mammary epithelial cells

Mammary tissues were obtained from 3 Chinese Holstein cows after their slaughter in a commercial cattle slaughter farm per time. The primary bovine mammary epithelial cells (pBMECs) were isolated and cultured by the adherent culture of small-sized cow mammary tissue. After mammary tissues were surgically removed from the slaughtered cow, they were placed in sterile, ice-cold phosphate-buffered saline (PBS) that was supplemented with 300 U/mL penicillin G and 100 mg/mL streptomycin (V900929, Sigma-Aldrich, Inc. St. Louis, MO, USA) and transported immediately to the laboratory. The mammary tissues were trimmed of visible fat and connective tissue and washed with PBS several times until the solution became pellucid and devoid of milk. Then, the mammary tissues were cut into small pieces (approx. 1×1×1 mm^3) and established as a primary culture, from which bovine mammary epithelial cells (BMECs) were isolated. The isolated cells were purified by differential digestion method with trypsin, and purified cells were identified. Cell morphology was examined by light microscopy.

1.2 Isolation of primary bovine mammary fibroblasts

The bovine mammary fibroblasts were isolated and cultured by the adherent culture of small-sized cow mammary tissue. The isolated cells were purified by differential digestion method with trypsin, and purified bovine mammary fibroblasts were identified as negative control cells for detecting *CSN2 and VIM (vimentin*) by RT-PCR with the primers (Table S1), and for examination of CSN2 and VIM (vimentin) by immunofluorescence. Cell morphology was examined by light microscopy.

1.3 Transcription of *KRT8*, *KRT18, CSN2* and *VIM* by RT-PCR in pBMECs and in bovine mammary fibroblasts

Total RNA was isolated using RNAzol (9109, TaKaRa Co. Ltd., Dalian, China)

from pBMECs and bovine mammary fibroblasts, respectively, and was reverse-transcribed with an oligo (dT)12–18 primer using the *EasyScript*® One-Step gDNA Removal and cDNA Synthesis SuperMix Kit (AE311, TransGen Biotech Co. Ltd., Beijing, China). The transcription of *KRT8*, *KRT18* and *CSN2* was detected by RT-PCR in pBMECs, and the transcription of *VIM*, *CSN2* was detected by RT-PCR in bovine mammary fibroblasts. An input of 1 μg total RNA was used for each reaction. cDNA sequences were amplified with the primers in Table S1. The RT-PCR program was as follows: 94°C for 3 min; 30 cycles at 94°C for 30 s, 55°C for 30 s, and 72°C for 1 min; and a final extension at 72°C for 10 min. The 10-μL PCR mixture contained 5 μL 2×*TransTaq*® HiFi PCR SuperMix II (AS131, TransGen Biotech Co. Ltd. Beijing, China), 0.5 μL 10 mM of each of the forward and reverse primers, 1 μL template cDNA, and 3 μL deionized water. The PCR products were electrophoresed, and photographs were taken on an UV transilluminator (UVItec, London, UK).

Table S1. The biomarker genes and primers for PCR

Gene Name	Primer sequence (5'→3')
KRT8 (NM_001033610.1)	P1: TGGAAGGGCTGACTGATGAG
	P2: GCTTCCTGTAGGTGGCAATC
KRT18 (NM_001192095.1)	P1: CAGGGCGAGAAGGAGACCAT
	P2: TAAGGTCCTGAGGTTTGGGG
CSN2 (XM_010806178.2)	P1: ATCCCTAACAGCCTCCCAC
	P2: AGAAAGGGACAGCACGGAC
VIM (NM_173969.3)	P1: CGCTTCGCCAACTACATC
	P2: TACCATTCCTCAGCCTCC

1.4 Expression of KRT7, KRT18, CSN2 and VIM (vimentin) by immunofluorescence in pBMECs and in bovine mammary fibroblasts

Primary BMECs and bovine mammary fibroblasts were seeded into 24-well plates at 1×10^5 cells per well, respectively, and incubated with culture medium until 80% confluence. Then, the cells were washed with PBS 3 times and fixed in 4% paraformaldehyde. Cells were treated for 5 min with 0.2% TritonX-100; 10% sheep blood was used to block the cells for 10 min. Next, the cells were incubated overnight at 4°C with the primary antibodies: anti-KRT7 (bs-1744R, Absin Bioscience,

Shanghai, China), anti-KRT18 (abs123946, Absin Bioscience, Shanghai, China), anti-CSN2 (sc-53189, Santa Cruz Biotechnology, Dallas, TX, USA), and anti-VIM (sc-58899, Santa Cruz Biotechnology, Dallas, TX, USA). Peroxidase-conjugated secondary antibody was added to the cells at room temperature for 1 h. The target proteins KRT7, KRT18 and CSN2 in pBMECs, and VIM and CSN2 in bovine mammary fibroblasts, were stained with SABC-Cy3 (SABC-Cy3 immunofluorescence staining kit, Rabbit, SA1074; Mouse, SA1072, Boster Biological Technology, Wuhan, China). The cells were incubated with SABC-Cy3 for 30 min at room temperature and imaged under a digital fluorescence microscope.

2. Results

Primary bovine mammary epithelial cells (pBMECs) and bovine mammary fibroblasts were isolated by the adherent culture of small-sized cow mammary tissues, respectively. The morphology of pBMECs was of a typical epithelial cell (Figure S1 (a), (b)), and the morphology of these fibroblasts was of a typical fibroblast with a spindle shape and several protrusions (Figure S1 (c)). The biomarker genes *KRT18*, *KRT8,* and *CSN2* were transcribed (Figure S1 (d)), and the biomarker proteins KRT7, KRT18, and CSN2 were expressed (Figure S1 (e)) in pBMECs. The biomarker gene *VIM* and protein VIM (vimentin), which is a biomarker of fibroblasts, were expressed, while *CSN2* and CSN2 was not expressed in bovine mammary fibroblasts (Figure S1 (f, g)).

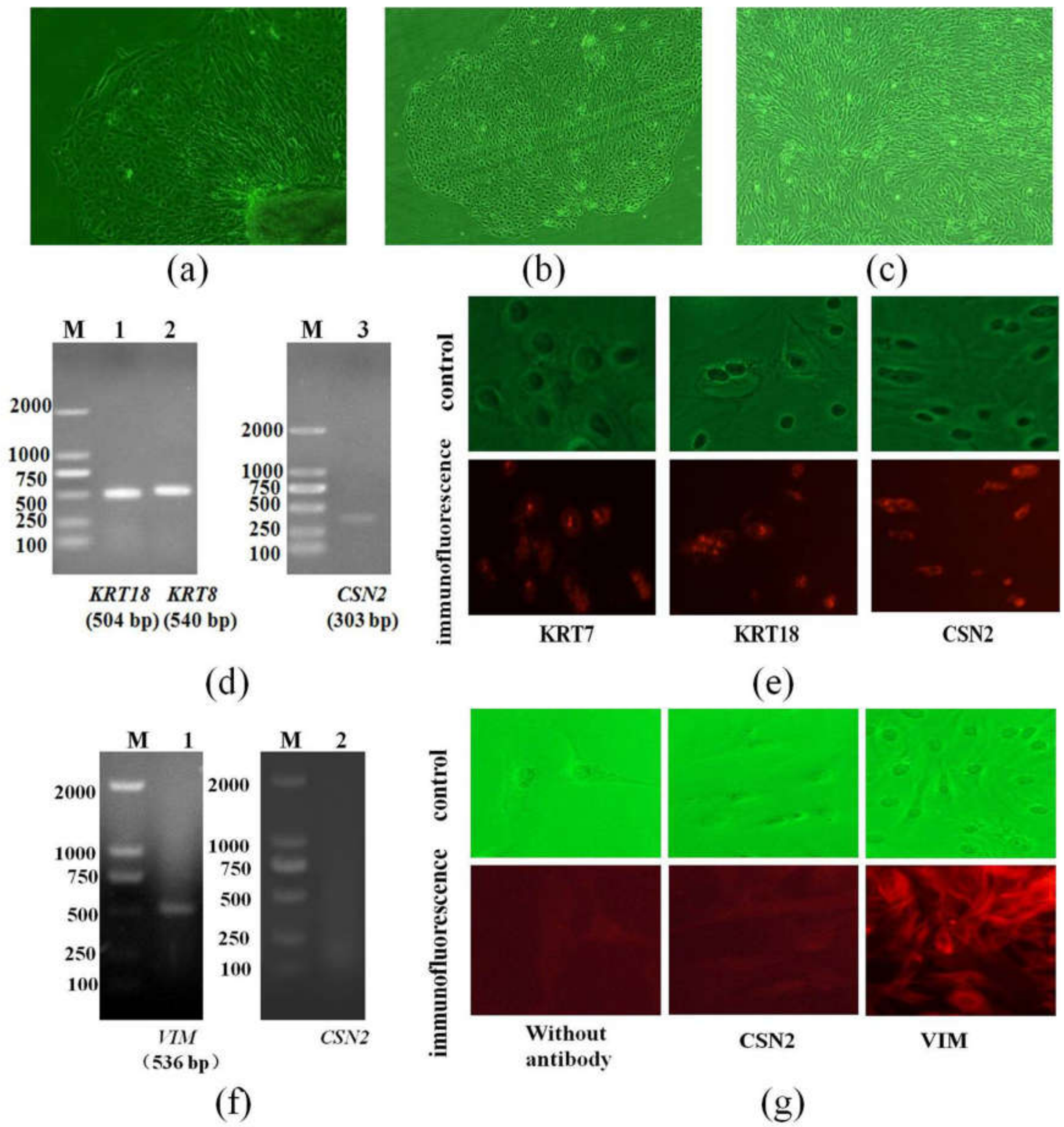

FIGURE S1. Morphology, biomarker genes, and biomarker proteins in primary bovine mammary epithelial cells

(a) Primary bovine mammary epithelial cells were isolated by the adherent culture of small-sized cow mammary tissue for 7 days.

(b) Purified bovine mammary epithelial cells.

(c) Purified bovine mammary fibroblasts.

(d) Specific genes were transcribed in pBMECs. M: DL2000 Marker; PCR products pg 1: of *KRT18* (504 bp); 2: *KRT8* (540 bp); and 3: *CSN2* (303 bp).

(e) KRT7, KRT18, and CSN2 were expressed in pBMECs.

(f) *VIM* was transcribed, and *CSN2* was not in bovine mammary fibroblasts. M:

DL2000 Marker; PCR products pg 1: of *VIM* (536 bp); 2: *CSN2*.

(g) VIM was expressed, and CSN2 was not in bovine mammary fibroblasts.

Supplementary File 2

Table S2 The internal control genes and primers for qPCR

Gene Name	Primer sequence (5'→3')
GAPDH (NM_001034034.2)	P1: CAGTCAAGGCAGAGAACG<br>P2: TTCACGCCCATCACAAAC
RPS15A (NM_001037443.2)	P1: CTCAAGAGTATCAACAATGCCG<br>P2: CCACACTTATTTAGCCTGCCTG
PPIA (NM_178320.2)	P1: GAGCACTGGAGAGAAAGGAT<br>P2: AAACTGGGAACCATTTGTGT
B2M (NM_173893.3)	P1: CCTTGGTCCTTCTCGGGC<br>P2: TAATCTTCTCCCCATTCTTCAG
ACTB (NM_173979.3)	P1:CACCACGGCCGAGCGGAAATC<br>P2:AGAGCCTCAGGGCAGCGGAACC

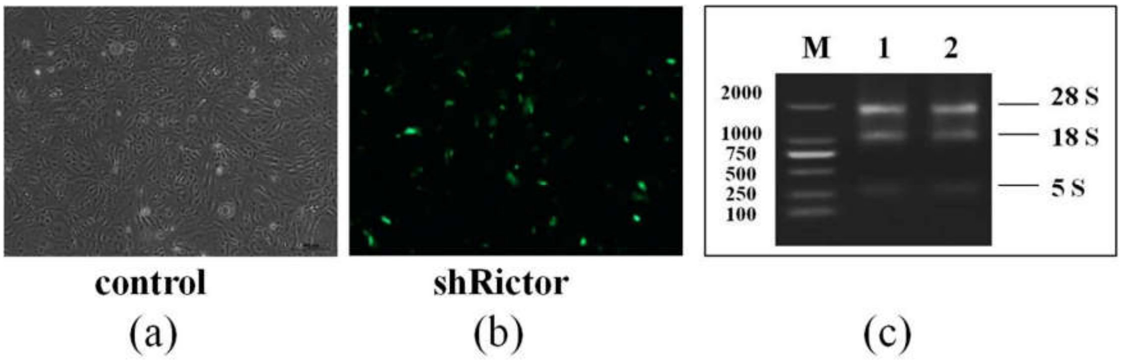

Figure S2. *RICTOR* knocked down using targeting shRNA.

(a) Control cells. (b) pRNAT-U6.1/Neo-RICTOR-shRNA-transfected pBMECs expressing the green fluorescent protein (EGFP). Scale bar: 200 px. (c) RNA integrity was assessed via electrophoresis. Total RNA was isolated from control cells and *RICTOR* silencing cells, respectively. RNA quantity of control cells was 883.5 ng/μL,

and the *RICTOR* silencing cells was 683.4 ng/μL. The RNA stock solution was diluted 10 times and then taken 2 μL for electrophoresis. Total RNAs pg M: DL2000 Marker; 1: of control cells; 2: *RICTOR* silencing cells.
